# Telomere DNA recognition in Saccharomycotina yeast: potential lessons for the co-evolution of ssDNA and dsDNA-binding proteins and their target sites

**DOI:** 10.3389/fgene.2015.00162

**Published:** 2015-05-01

**Authors:** Olga Steinberg-Neifach, Neal F. Lue

**Affiliations:** ^1^Department of Microbiology and Immunology, W. R. Hearst Microbiology Research Center, Weill Medical College, Cornell University, New York, NY, USA; ^2^Hostos Community College, City University of New York, Bronx, NY, USA

**Keywords:** telomere, telomere-binding proteins, Saccharomycotina, co-evolution of DNA and binding proteins, gene duplication, dimerization, Rap1, Cdc13

## Abstract

In principle, alterations in the telomere repeat sequence would be expected to disrupt the protective nucleoprotein complexes that confer stability to chromosome ends, and hence relatively rare events in evolution. Indeed, numerous organisms in diverse phyla share a canonical 6 bp telomere repeat unit (5′-TTAGGG-3′/5′-CCCTAA-3′), suggesting common descent from an ancestor that carries this particular repeat. All the more remarkable, then, are the extraordinarily divergent telomere sequences that populate the Saccharomycotina subphylum of budding yeast. These sequences are distinguished from the canonical telomere repeat in being long, occasionally degenerate, and frequently non-G/C-rich. Despite the divergent telomere repeat sequences, studies to date indicate that the same families of single-strand and double-strand telomere binding proteins (i.e., the Cdc13 and Rap1 families) are responsible for telomere protection in Saccharomycotina yeast. The recognition mechanisms of the protein family members therefore offer an informative paradigm for understanding the co-evolution of DNA-binding proteins and the cognate target sequences. Existing data suggest three potential, inter-related solutions to the DNA recognition problem: (i) duplication of the recognition protein and functional modification; (ii) combinatorial recognition of target site; and (iii) flexibility of the recognition surfaces of the DNA-binding proteins to adopt alternative conformations. Evidence in support of these solutions and the relevance of these solutions to other DNA-protein regulatory systems are discussed.

## Overview

Linear eukaryotic chromosome termini are stabilized by telomeres, which are specialized nucleoprotein complexes that suppress the recognition of the ends as double strand breaks (DSBs; de Lange, 2009; O’Sullivan and Karlseder, 2010; Jain and Cooper, 2011). This stabilization is mediated by a collection of telomeric proteins that associate directly or indirectly with the repetitive telomeric DNAs and that suppress the action of checkpoint and repair proteins. The DNA component of telomeres typically comprises a duplex region of hundreds to thousands of nucleotides and a 3′ overhang of tens to hundreds of nucleotides (referred to as the G-tail because of its G-rich nucleotide composition). Both the duplex region and 3′ overhang are comprised of the same short repeat unit, and both are bound by sequence-specific recognition proteins, which in turn recruit other proteins crucial for telomere protection. Because proteins recruited to the duplex telomere repeats and G-tails are both required for telomere stability, the duplex/G-tail DNA structural arrangement at chromosome ends is evidently essential for telomere function. Besides telomere protection, the other major function of telomere-bound proteins is to maintain telomere DNAs. Despite their fundamental importance, telomere DNAs can experience progressive loss owing to incomplete end replication (Olovnikov, 1996), as well as drastic truncation owing to recombinational excision or replication fork collapse (Lustig, 2003; Lansdorp, 2005). To compensate for such losses, eukaryotic cells employ telomerase and the primase-pol α complex to extend the G-tail and the complementary C-strand of telomeres, respectively (Autexier and Lue, 2006; Blackburn and Collins, 2011; Nandakumar and Cech, 2013; Pfeiffer and Lingner, 2013; Lue et al., 2014). Not surprisingly, these telomere extension activities are subjected to elaborate control by telomere-bound proteins in order to maintain telomere lengths within a size range that is appropriate for telomere function.

**FIGURE 1:**
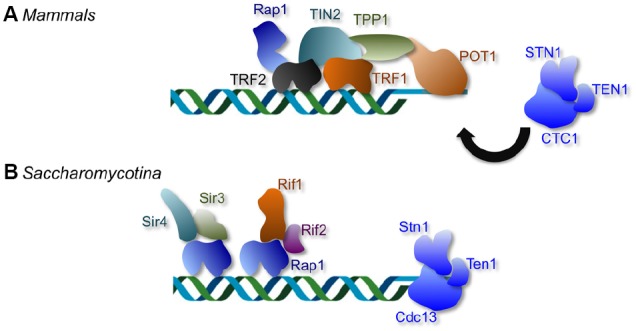
**The distinctive telomere protective complexes in mammals and in Saccharomycotina yeast. (A)** The mammalian telomeres are bound by a six-protein complex collectively named shelterin. Within the telomere nucleoprotein complex, duplex telomeres are bound directly by TRF1 and TRF2, and G-tails are bound directly by POT1. The mammalian CST (CTC1-STN1-TEN1) complex plays minimal roles in telomere protection, but is crucial for regulating telomere DNA synthesis. **(B)** The telomere complexes of Saccharomycotina yeast display considerable differences from those in other phyla; the duplex telomeres and G-tails in Saccharomycotina yeast are bound by Rap1 and Cdc13, respectively. Like CTC1, the fungal Cdc13 is part of the CST complex that also contains Stn1 and Ten1. However, unlike mammalian CST, the fungal CST complex is crucial both for telomere protection and for regulating telomere DNA synthesis.

A particularly prevalent telomere repeat unit, found in various fungi, plant, metazoans, and protozoa, is 5′-TTAGGG-3′/5′-CCCTAA-3′. In organisms with this telomere repeat unit, the duplex region is typically recognized directly by a member of the telomere repeat binding factor (TRF) protein family, whereas the 3′ overhang bound directly by that of the protection of telomeres 1 (POT1) protein family (Figure 1). In most mammalian cells, for example, two TRF homologs (TRF1 and TRF2) and a POT1 homolog constitute the three direct DNA-binding components of the six-protein “shelterin” complex that collectively protects the duplex telomeres and G-tails (Figure 1; de Lange, 2009). In fission yeast, on the other hand, a single TRF homolog (Taz1) and a POT1 homolog (Pot1) account for direct DNA-binding by a somewhat different version of the shelterin complex (Jain and Cooper, 2011). Both the TRF and POT1 family members have been subjected to extensive structural and functional investigations, and the molecular bases of their DNA recognition mechanisms are understood at the level of atomic resolution structures (Fairall et al., 2001; Lei et al., 2003, 2004; Court et al., 2005). TRF proteins form homodimers through their N-terminal TRF homology (TRFH) domain, and use the resulting tandem C-terminal Myb DNA-binding domains (DBDs) to make contacts with two adjacent repeat units. POT1 uses a pair of OB (oligonucleotide/oligosaccharide binding) folds to interact with ∼10 nt of the G-rich, 3′ end containing strand of telomeres [i.e., the (TTAGGG)n strand]. Sequence recognition by both proteins is highly specific such that most nucleotide substitutions in the target DNA cause a substantial loss in binding affinity. This sequence specificity is to be expected: given the capacity of the telomere proteins to “stabilize” DNA ends and prevent recombination and end-joining, promiscuous binding of these proteins to DNA DSBs would presumably be detrimental to the cells.

Implicit in the foregoing discussion are the substantial constraints imposed on the telomere nucleoprotein system during evolution. The greater constraints of the telomere system are evident when one compares its parts to those of a more circumscribed system consisting of, e.g., a transcription factor and its target site. In the latter case, a point mutation in the DNA target site could be readily accommodated by perhaps a few changes in the transcription factor DNA-binding surface. However, a comparable point mutation in the canonical telomere repeat unit is likely to cause greater disruption of cellular function and require greater compensatory adjustments. Loss of TRF or POT1 binding to the mutated repeat will probably cause extensive changes in the chromatin structure of telomeres. Conversely, restoration of normal telomere structure in this setting may require multiple changes in the binding surfaces of both TRF and POT1. Viewed in this light, it is perhaps not surprising that numerous present-day organisms in diverse phyla have retained the canonical, presumably ancient telomere repeat sequence and TRF and POT1 homologs. Examples of such organisms include fungi (e.g., basidiomycotina and pezizomycotina), metazoans (e.g., vertebrates), plants (e.g., *Aloe sp*., *Hyacinthella dalmatica*, and *Othocallis siberica*), and even protists (e.g., trypanosome and *Leishmania*), where the TTAGGG repeat is relatively uncommon (Podlevsky et al., 2008).

## The Unusual Telomere Repeats of Saccharomycotina Fungi

One group of organisms with telomere systems that deviate from the canonical system is found in the Saccharomycotina subphylum of budding yeast (Figure 1). They include a widely used model organism, several pathogenic fungi, and others (*Saccharomyces*, *Kluyveromyces*, and *Candida*). The telomere repeats in these organisms are extraordinarily divergent and differ from the canonical repeat in being long, occasionally degenerate, and frequently non-G/C-rich. Notably, the telomeres of Saccharomycotina yeast are not bound directly by TRF and POT1 family members, but rather by two other distinct protein families named Rap1 and Cdc13, suggesting that the acquisition of atypical telomere DNA sequences was accompanied by a substantial remodeling of the telomere nucleoprotein structure (Figure 1). Remarkably, homologs or structural equivalents of Rap1 and Cdc13 also exist in organisms with the canonical telomere repeat sequence, but these homologs or equivalents clearly mediate distinct functions in these organisms. Mammalian RAP1, while a component of the shelterin complex, exhibits low affinity for telomere repeats and is localized to telomeres primarily through an interaction with TRF2 (Li et al., 2000; Arat and Griffith, 2012). The mammalian equivalent of Cdc13, named CTC1, is like Cdc13, a component of the CST (CTC1-STN1-TEN1) complex that also contains Stn1 and Ten1 (Miyake et al., 2009; Surovtseva et al., 2009). However, unlike Cdc13, CTC1 has little function in telomere protection, and appears to be primarily involved in regulating telomere DNA synthesis (Price et al., 2010; Stewart et al., 2012). The existence of mammalian CTC1 and RAP1 strongly suggests that fungal Cdc13 and Rap1 were not acquired *de novo*, but were co-opted to perform a new telomere function (i.e., direct telomere DNA-binding) as a pre-existing telomere component. Evolutionary models that account for the transition from the canonical telomere architecture to that found in Saccharomycotina yeast have been presented before, and will not be re-iterated in this review (Lue, 2010). Instead, we focus our discussion on a major evolutionary conundrum presented by the telomeres of this group of fungi, i.e., the DNA recognition challenge posed by rapidly evolving telomere sequence.

Interestingly, even though Rap1 exhibits little sequence similarity to TRF and has a distinct domain organization, it also utilizes Myb-like homeodomains for telomere DNA-binding. Likewise, Cdc13 can hardly be aligned to POT1 at the sequence level, yet both protein families employ the same OB fold scaffold for single-strand DNA (ssDNA) recognition. Unlike TRF and POT1, however, fungal Rap1s and Cdc13s are tasked with recognizing a very diverse collection of telomere target sequences. According to the estimates of evolutionary models, the Saccharomycotina yeasts share a common ancestor as recently as 300 million years ago, and yet collectively possess more than 20 distinct telomere repeats (Pesole et al., 1995; Hedges, 2002). *A priori*, this degree of evolutionary divergence can only be considered highly unusual. In terms of coding sequences, the *Candida* and *Saccharomyces* genomes are approximately as divergent as those of fish and humans, which possess the same canonical telomere sequence (Dujon et al., 2004). How then, do the major double-strand (ds) and ss telomere binding proteins (i.e., Rap1 and Cdc13) acquire the correct sequence-specificity for the rapidly changing telomere sequence? Even though we are far from having a complete answer, recent studies suggest a number of solutions to this challenge. In the following sections, we discuss in detail the structure, function and evolution of Rap1 and Cdc13, with a special emphasis on their evolutionary plasticity and their versatile DNA binding mechanisms that enables them to adapt to the multiplicity of target sequences. (In discussing the target sequence of Rap1, we will refer to the G-strand sequence such that the same strand is used in describing both the Rap1 and Cdc13 targets. This is in contrast to the majority of previous articles that characterize Rap1 binding sites.)

## Rap1

**FIGURE 2:**
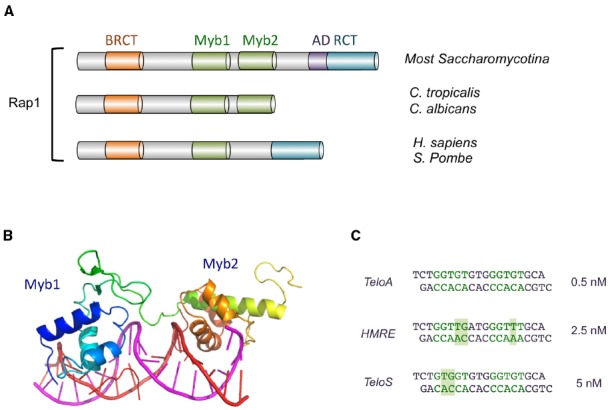
**The domain organization of Rap1 and the structure of Rap1_DBD_-DNA complex. (A)** The domain structures of Rap1 from various Saccharomycotina and other species are illustrated. The BRCT, Myb, AD (activation domain), and RCT (Rap1 C-terminal) domains are displayed in different colors. **(B)** The crystal structure of the Myb1 and Myb2 domains of *S. cerevisiae* Rap1 (shown in color spectrum from blue to orange) bound to its target DNA (shown in magenta and red; PDB ID: 1IGN). **(C)** The sequences of the three duplex oligonucleotides bound by *Sc*Rap1_DBD_ in a crystallographic study are displayed. The half sites in each oligo are shown in green, and nucleotides that deviate from a canonical half site (5′-GGTGT-3′/5′-ACACC-3′) are shown with a shaded background. The affinities of Rap1_DBD_ for each sequence are shown on the right. Other variant targets (e.g., the site upstream of ribosomal protein genes: AAATGTATGGGTGT) have been reported to have comparable affinities (Idrissi et al., 1998; Idrissi and Pina, 1999).

Rap1 (Repressor activator protein 1, also originally known as GRF1 or TUF1), a conserved telomere protection factor, exhibit remarkable functional versatility (Shore, 1994). Notably, it was first discovered in *Saccharomyces cerevisiae* as a transcriptional regulator of numerous metabolic genes (Huet et al., 1985). Subsequent studies implicate Rap1 as a key component of the mating type silencer as well as the major ds telomere DNA binding protein (Shore et al., 1987; Buchman et al., 1988). That a single factor mediates such diverse functions at distinct chromosomal locations certainly raises interesting mechanistic and evolutionary issues that remain incompletely resolved. The multi-functional nature of Rap1 is evidently conserved in evolution; mammalian Rap1 has also been reported to regulate transcription and protect telomeres (Li et al., 2000; Martinez et al., 2010; Sfeir et al., 2010). However, a recent study suggests that the telomere protection function of human Rap1 may be quite minor and perhaps non-existent (Kabir et al., 2014). At telomeres, Rap1 displays striking malleability by interacting with different molecular targets in different organisms. In budding yeast, Rap1 binds ds telomere DNAs directly with high affinity and sequence specificity, whereas in fission yeast and mammals (and probably most other organisms), Rap1 is recruited to telomeres through interaction with other telomere proteins such as TRF2 and Taz1 (Li et al., 2000; Kanoh and Ishikawa, 2001). In keeping with its multi-functional nature, *S. cerevisiae* Rap1 possesses a complex domain organization (Figure 2A). Near its N-terminus is a BRCA1 C-terminus (BRCT) domain, a presumed protein interaction domain whose targets may include Gcr1, another transcription factor (Lopez et al., 1998). Located centrally is the DBD, which uses a pair of Myb motifs to interact with DNA (Giraldo and Rhodes, 1994; Wahlin and Cohn, 2000; Figures 2A,B). At the C-terminal end of Rap1 is a purely alpha helical structure Rap1 C-terminus (RCT) that has been shown to mediate interactions with other proteins required for proper telomere structure and function (e.g., Sir3, Sir4, Rif1, and Rif2; Feeser and Wolberger, 2008). Finally, a region between the DBD and RCT has been ascribed a transcriptional activation function (Shore, 1994). With a few exceptions (e.g., *C. albicans* Rap1 lacks RCT) this domain organization is conserved in other Saccharomycotina homologs. However, fission yeast and mammalian Rap1s display structural and functional differences, owing perhaps to their different means of telomere localization; these Rap1s carry a single Myb motif that binds DNA with low affinity, and an RCT that tethers Rap1 to a high-affinity DNA-binding protein (i.e., Taz1 in *S. pombe* and TRF2 in mammals; Li et al., 2000; Kanoh and Ishikawa, 2001; Arat and Griffith, 2012; Figures 1 and 2A).

The DNA-binding activity of the Rap1 DBD was first characterized for the *S. cerevisiae* protein, and the binding of *Sc*Rap1 to numerous DNA targets (∼200–300 promoters, two silencers, and several telomeric variants) have been investigated individually and at genome-wide scale (Idrissi and Pina, 1999; Lieb et al., 2001; Pina et al., 2003; Yarragudi et al., 2007; Rhee and Pugh, 2011). While several consensus sequences for Rap1 have been reported, a frequently noted version is K_13′_R_12′_T_11′_G_10′_T_9′_R_8′_Y_7′_G_6′_G_5′_G_4′_T_3′_G_2′_T_1′_ (Lieb et al., 2001). This somewhat degenerate consensus consists of two half sites, K_13′_R_12′_T_11′_G_10′_T_9′_ and G_5′_G_4′_T_3′_G_2′_T_1′_, bound respectively by the second and first Myb motif in Rap1. Subsets of Rap1 targets (e.g., at ribosomal protein gene promoters) exhibit distinctive features with regard to their sequences and dispositions, suggesting that the activities of Rap1 at different chromosomal locations may be modulated by its binding to specific variants of the consensus, i.e., Rap1 may adopt different conformations, and hence recruit different co-factors depending on the specific target sequence to which it is bound (Pina et al., 2003).

As implied from the foregoing discussion, *Sc*Rap1 displays considerable flexibility in recognizing diverse target site sequences. This flexibility stems in part from the ability of the Myb motifs to tolerate many variations in the target sequence (especially the half site comprised of residues 13′–9′) without suffering a loss in binding affinity (Vignais et al., 1990; Idrissi and Pina, 1999). This is evident from the loose consensus reported for Rap1, and especially the more degenerate sequence reported for the first half site. The molecular basis for the flexibility of Rap1 has been investigated through crystallographic analysis of three complexes formed between *Sc*Rap1_DBD_ and different DNA target sites (Figure 2C; Konig et al., 1996; Taylor et al., 2000). Overall, the results indicate that recognition of base pairs that vary between the target sites is accomplished through the utilization of alternative side-chain conformations and alternative contacts to the nucleotides. In other words, rather than altering its overall configuration, Rap1 modifies its fine surface structure to suit the demand of a particular target sequence. This inherent versatility is not unique to Rap1 (see, e.g., Schwabe et al., 1995), but appears to be highly developed in this protein, and may have allowed it to handle the challenge presented by the rapidly evolving telomere sequence in Saccharomycotina yeast (see below).

Another (probably minor) source of flexibility may be the number of nucleotides that separate the two half sites. In the vast majority of well-characterized target sites, this number is three such that the center-to-center distance between the two half sites is 8 bp (Pina et al., 2003). However, in a footprinting analysis utilizing a variant telomere sequence derived from *S. castellii*, *Sc*Rap1 produced a split footprint indicative of a center-to-center distance of 14 nt, suggesting that an atypical separation between the half sites can be tolerated in rare cases, possibly through looping out of the intervening DNA (Wahlin and Cohn, 2000).

**FIGURE 3:**
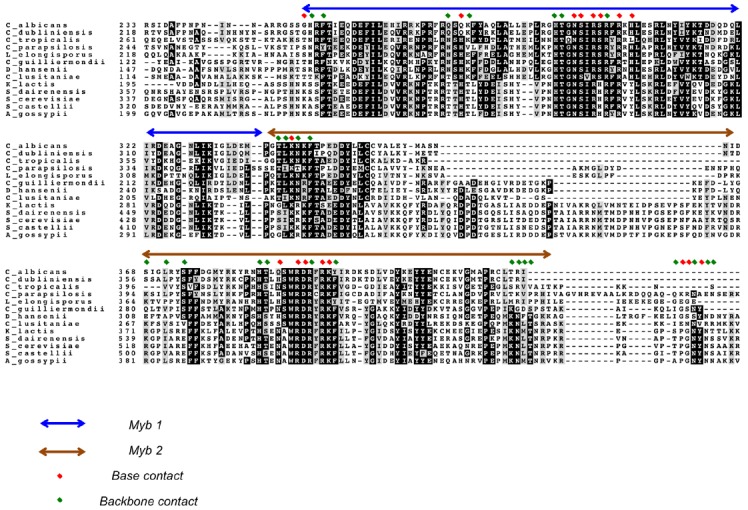
**An alignment of Rap1_DBDs_ showing the variable extent of sequence conservation of residues implicated in DNA contacts.** An alignment of the DBDs of selected Saccharomycotina Rap1 homologs is displayed. The first and second Myb motifs are indicated by blue and brown arrows, respectively. Residues implicated in making base and backbone contacts are designated by red and green diamonds, respectively. Dark and gray shading of amino acids are used to highlight strict conservation and conservative substitution, respectively.

Because all Saccharomycotina Rap1 homologs possess duplicated Myb motifs, it seems likely they all use such motif pairs for direct DNA-binding. This proposition is consistent with studies of two Rap1 family members, namely those in *S. castellii* and *C. albicans*. Specifically, the pairs of Myb motifs in each protein alone have been shown to be just as active in DNA-binding as the respective full-length protein (Wahlin and Cohn, 2002; Yu et al., 2010; Rhodin Edso et al., 2011). While not as well characterized as *Sc*Rap1, the DNA-binding mechanisms of *Scas*Rap1 and *Ca*Rap1 also appear to be quite similar to that for *Sc*Rap1 with respect to target site arrangement and sequence. For *Scas*Rap1, the minimal high affinity target is a 12-bp duplex (GGGTGTCTGGGT), within which just three positions (G1, C7, T12) appear to have non-stringent sequence requirement (Rhodin Edso et al., 2011). For *Ca*Rap1, the high affinity target consists of two 5-bp elements (GGTGT and GGATG) separated by two base pairs of random nucleotides (Yu et al., 2010). These observations are quite consistent with the notion of consecutive Myb motifs each recognizing 4–5 bp of G-rich elements. The exact identity of the first half site (GGTGT), which is the target of the second Myb motif according to the *Sc*Rap1_DBD_-DNA crystal structure, suggests that the mechanisms of this second Myb motif in telomere DNA-binding may be quite well conserved in evolution. On the other hand, the half-site separations for *Scas*Rap1 and *Ca*Rap1 appear to be smaller than, and the consensus sequences for their second half sites quite different from that of *Sc*Rap1, consistent with significant adaptation of these Rap1 orthologs to their cognate telomere sequences. Notably, residues in the first Myb motif of *Sc*Rap1 implicated in direct base contact appear to exhibit greater sequence variation among all the Saccharomycotina homologs than comparable residues in the second Myb motif (Figure 3). This difference could reflect adaption of the first Myb to the more divergent target sites (i.e., the second half site). A notable difference between *Candida* and *Saccharomyces* Rap1s is that the former has a far less significant role in transcriptional regulation and does not appear to bind to the promoters of many metabolism-related genes (Lavoie et al., 2010; Yu et al., 2010). Hence, it is unclear if *Ca*Rap1 possesses the same degree of target site recognition versatility as that possessed by *Sc*Rap1. Nevertheless, the versatility exhibited by *Sc*Rap1 indicates that members of this protein family has a variety of means to bind alternative sequences, and hence is well positioned to handle the challenge posed by the rapidly evolving telomere sequence in Saccharomycotina yeast.

## Cdc13

Cdc13 (cell division cycle 13), the major G-tail binding protein in Saccharomycotina yeast, is like Rap1, a multifunctional protein with a complex domain organization (for reviews, see Giraud-Panis et al., 2010; Lue, 2010). As the name implies, it was initially characterized as a gene in *S. cerevisiae* that when mutated, causes cell cycle defects (Garvik et al., 1995). Subsequent studies uncovered not only the G-tail binding activity of *Sc*Cdc13, but also multiple functions for this protein at telomeres, including protecting telomeres against C-strand degradation, as well as regulation of both telomerase and Pol α in their telomere DNA synthesis activities (Nugent et al., 1996; Qi and Zakian, 2000; Pennock et al., 2001). For a subset of these functions, *Sc*Cdc13 works as part of a complex (CST) that also contains Stn1 and Ten1 (Giraud-Panis et al., 2010).

**FIGURE 4:**
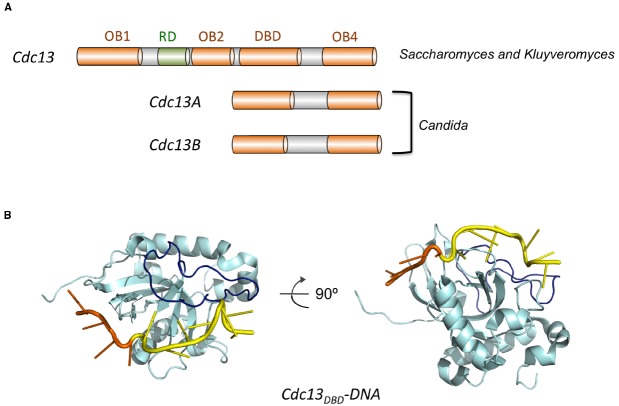
**The domain organization of Cdc13 and the structure of Cdc13_DBD_-DNA complex. (A)** The domain structures of Cdc13 homologs from various Saccharomycotina species are illustrated. The four OB folds (OB1, OB2, DBD, and OB4) are displayed in orange, and the RD (recruitment domain) is shown in green. **(B)** The structure of the DBD of *S. cerevisiae* Cdc13 bound to its target DNA (PDB ID: 1S40). Cdc13 is shown in cyan with the L23 loop highlighted in dark blue. The 5′ four nucleotides of the DNA target is shown in orange and the 3′ seven nucleotides shown in yellow.

Structurally, *Sc*Cdc13 is quite large (924 aa) and complex, and is comprised of four OB fold domains that bind distinct molecular targets to mediate telomere protection and maintenance (Figure 4A). *Sc*Cdc13_OB1_ forms dimers to create a binding groove for Pol1 (the catalytic subunit of DNA polymerase α), and may possess a low affinity G-strand-binding activity as well as binding sites for other proteins (Hsu et al., 2004; Mitchell et al., 2010; Sun et al., 2011). *Sc*Cdc13_OB2_ also forms dimers and modulates interaction between Cdc13 and Stn1 (Mason et al., 2012). The third OB fold (*Sc*Cdc13_DBD_) constitutes the high affinity G-strand-binding domain, and the final OB fold (*Sc*Cdc13_OB4_) mediates interaction with Stn1 (Hughes et al., 2000; Sun et al., 2011; Yu et al., 2012). In addition to these OB fold domains, Cdc13 also carries a telomerase recruitment domain (RD) that binds to the telomerase regulatory subunit Est1 and that is required for telomere localization of telomerase (Pennock et al., 2001; Wu and Zakian, 2011).

Interestingly, analysis of other Cdc13s in Saccharomycotina yeast revealed a high degree of structural malleability and evolutionary plasticity. While all *Saccharomyces* and *Kluyveromyces spp.* carry just one Cdc13 homolog that resembles structurally *Sc*Cdc13, most *Candida spp*. carry two Cdc13 homologs (named Cdc13A and Cdc13B), each containing just two OB fold domains that align well to *Sc*Cdc13_DBD_ and *Sc*Cdc13_OB4_ (Figure 4A). The accumulated structural and functional evidence suggests that *Sc*Cdc13 (and other large Cdc13s) may arise through a fusion of Cdc13A and Cdc13B in the common ancestor of Saccharomycotina yeast (Lue and Chan, 2013).

The G-tail binding activity of Cdc13 was first characterized (not surprisingly) for the *S. cerevisiae* protein. Proteolytic and deletion analyses defined a stable domain (*Sc*Cdc13_DBD_, amino acid 557 to 692) that exhibits high affinity (sub nanomolar) for a variety of target sites that correspond to different variants of the irregular *Sc* G-strand repeats (G_1–3_T; Hughes et al., 2000; Anderson et al., 2003). Even though full length *Sc*Cdc13 is naturally dimeric, the DBD domain behaves as a monomer in solution and binds DNA as a monomer. The minimal size for high affinity ligands is reported to be ∼11 nt, and the affinities of *Sc*Cdc13_DBD_ for these ligands are typically similar to or better than those of full length *Sc*Cdc13. Nuclear magnetic resonance (NMR) investigations of *Sc*Cdc13_DBD_ revealed an OB fold structure, which is quite common for ss nucleic acid-binding proteins (Mitton-Fry et al., 2002, 2004; Figure 4B). A structural motif shared by numerous proteins, the OB fold is comprised of five beta strands (S1 through S5) that adopt the shape of a miniaturized barrel (Theobald et al., 2003; Bochkarev and Bochkareva, 2004). For most ssDNA-binding OB folds, residues in L12 (the loop connecting S1 and S2), L45 and the central beta strand (S3) are typically responsible for contacting a short (4–6 nt) ligand. A standard polarity prevails in the vast majority of OB-ssDNA complexes such that L45 and L12 interact with the 5′ and 3′ portion of the target site, respectively. A distinctive feature of *Sc*Cdc13_DBD_ is the presence of an extended and structurally well-defined L23 that makes contacts to nucleotides 3′ to the typical target site, thus expanding the ssDNA ligand to 11 nt (Mitton-Fry et al., 2004; Eldridge and Wuttke, 2008; Figure 4B). A combination of structural, biophysical and biochemical investigations have provided rich insights on the recognition mechanism of *Sc*Cdc13_DBD_ for an 11-nt high affinity ligand (GTGTGGGTGTG; K_d_ = 3 pM; Anderson et al., 2003; Mitton-Fry et al., 2004; Eldridge et al., 2006; Eldridge and Wuttke, 2008). Like many other ssDNA and RNA-binding proteins, the hydrophobic and aromatic residues in *Sc*Cdc13_DBD_ evidently make greater contribution to affinity than charged residues (Anderson et al., 2003). While amino acids that contribute significantly to binding can be identified throughout the DNA-protein interface, the most critical ones all interact primarily with the 5′-most four nucleotides (GTGT; Anderson et al., 2003; Mitton-Fry et al., 2004). The region surrounding the 5′ nucleotides appear to undergo conformational re-structuring upon DNA-binding, arguing for an induced fit mechanism that may enhance the specificity of interaction (Eldridge and Wuttke, 2008). In contrast, the 3′ nucleotides are bound chiefly by the extended L23 with less sequence specificity, which may allow *Sc*Cdc13_DBD_ to interact optimally with the heterogeneous *S. cerevisiae* telomere repeats (Eldridge and Wuttke, 2008).

In addition to *Sc*Cdc13, several other family members in the *Saccharomyces* and *Candida* lineages have been investigated with respect to their DNA-binding properties, revealing interesting mechanistic variations in the recognition of G-tails. *Scas*Cdc13 is comparable in size to *Sc*Cdc13, but possesses a functional DBD domain that is more extended on the N-terminal side by ∼70 aa (Rhodin Edso et al., 2008). The structural basis for this additional requirement is not understood. Although the affinity of *Scas*Cdc13 for the cognate G-tail has not been determined quantitatively, the DBD domain appears to possess an affinity similar to that of the full length protein (Rhodin Edso et al., 2008). The 8 bp minimal target site (GTGTCTGG) is somewhat smaller than the 11 nt target site for *Sc*Cdc13, and the most critical nucleotide residues (positions 3, 4, 7, and 8) do not cluster near the 5′ end, suggesting substantial differences in the mechanism of binding (even though the GT-rich nature of the target site is conserved).

As described earlier, instead of carrying a large, 4-OB Cdc13, each *Candida* spp. possesses two Cdc13 homologs (Cdc13A and Cdc13B), both of which contain just 2 OB folds. Despite their small size, the *Candida* Cdc13s are clearly orthologs of the large 4-OB Cdc13s. Like the 4-OB Cdc13s, the *Candida* homologs are enriched at telomeres, and are required for normal telomere structure and function (Lue and Chan, 2013). Sequence alignments suggest that the small Cdc13s are structurally similar to the C-terminal half of the large Cdc13s, i.e., they consist of just the DBD and OB4 domains. In addition to the size difference, the small Cdc13s also exhibit distinct dimerization properties; whereas the large Cdc13s utilize OB1 for stable dimerization, the small Cdc13s appear to use primarily OB4 for this purpose (Yu et al., 2012; Lue and Chan, 2013). Moreover, in the two species for which both Cdc13A and Cdc13B dimerization have been subjected to detailed analysis, the two paralogs appear to form preferentially heterodimers rather than homodimers (Lue and Chan, 2013; Steinberg-Neifach et al., 2015). Perhaps most interestingly, unlike ScCdc13, which uses a DBD monomer to mediate high affinity binding to G-tails, the *Candida* Cdc13s evidently require protein dimerization to achieve high affinity binding (Yu et al., 2012; Lue and Chan, 2013; Steinberg-Neifach et al., 2015).

The first *Candida* Cdc13 complex to be subjected to detailed DNA-binding analysis is the *C. tropicalis* Cdc13AA homodimer (Yu et al., 2012). (This analysis was performed prior to the discovery of *Ct*Cdc13B, and the activities of the *Ct*Cdc13AB and BB dimer, if any, remain uncharacterized.) Investigation of *Ct*Cdc13AA revealed two unexpected features. First, unlike both *Sc*Cdc13 and *Scas*Cdc13, the DBD domain of *Ct*Cdc13A alone is incapable of high affinity binding to the cognate G-tail. Instead, the formation of a stable DNA-protein complex requires dimerization of full length *Ct*Cdc13A mediated by the OB4 domain (Yu et al., 2012). Second, in keeping with the dimerization requirement, the high affinity DNA ligand consists of two copies of a 6-nt element (GGATGT) found within the *C. tropicalis* G-strand repeat unit. In the native *Ct* G-tail, the 6-nt elements are separated from one another by 17-nt, resulting in a minimal high affinity ligand (29-nt) that is far longer than those for *Sc*Cdc13 and *Scas*Cdc13. Additional characterization revealed substantial spatial flexibility between the two 6-nt elements in the high affinity complex: the distance can be as short as 10 nt (Yu et al., 2012). Thus, the individual DBDs of *Ct*Cdc13A evidently possess low affinity for a short ligand within the telomere repeat unit, requiring a pair of protein-ligand interactions conferred by the full length protein dimer to achieve high affinity Binding to G-tails.

As noted before, emerging data suggest that the *Candida* Cdc13s may exist preferentially as heterodimers, thus begging the question as to the recognition mechanism of this dimeric complex. This was first assessed in *Candida albicans* (Lue and Chan, 2013). Analysis of the *C. albicans* homodimers and heterodimers revealed substantial G-tail binding activities for both the AA and AB complex, but not the BB complex (Lue and Chan, 2013). However, the ligand requirements for *Ca*Cdc13AA and AB were not examine in detail due to the propensity of these complexes to form large aggregates. The second Cdc13 heterodimer to be analyzed was from *C. parapsilosis* (Steinberg-Neifach et al., 2015). Similar to *C. albicans*, the Cdc13 paralogs in *C. parapsilosis* can form homo-oligomeric complexes as well as heterodimers. Surprisingly, only the *Cp*Cdc13AB heterodimer exhibits robust G-tail binding activity. In contrast to *Ct*Cdc13AA, the formation of high affinity *Cp*Cdc13AB-DNA complex requires just one copy of the 6-nt consensus element. Additional studies revealed a minimal target site of ∼17 nt comprised of the 6-nt element and 11 nt on the immediate 5′ side of the element. Detailed investigation of the sequence specificity coupled with site-specific crosslinking assays uncovered an unprecedented “combinatorial” mechanism of G-tail recognition. In this mode of recognition, the DBDs of *Cp*Cdc13A and *Cp*Cdc13B make contacts to the 3′ and 5′ region of the repeat unit, respectively. Recognitions of both regions of the repeat are highly sequence-specific, thus enabling *Cp*Cdc13AB to bind its cognate target with much greater species-specificity than the *Ct*Cdc13AA complex. In addition, the OB4 domains of *Cp*Cdc13A and *Cp*Cdc13B contribute to high affinity binding by forming a stable heterodimer to promote the dimerization of the DBDs. These results indicate that in some *Candida spp*., the challenge of binding variant G-tails is met through the duplication of Cdc13, the hetero-dimerization of the paralogs, and the adaption of the DBDs to new target sequences. Studies of other additional *Candida* Cdc13s should provide insights on the general applicability of this proposal.

## Shared and Distinctive Features of ds and ss Telomere DNA Recognition in Saccharomycotina Yeast

As noted before, a unique attribute of the telomere system from the evolutionary standpoint is the need to maintain adequate recognition of the telomere DNA in both its double-stranded and single-stranded forms upon changes in the sequence of the DNA. The remarkable divergence of telomere repeat sequences in Saccharomycotina yeast indicates that the Rap1 and Cdc13 protein families are sufficiently versatile and malleable to meet the challenge. While the mechanisms used by each family for DNA recognition are clearly distinct, some general themes can nevertheless be discerned. Below I list common and distinctive strategies utilized by these protein families to enable recognition of diverse sequence targets by family members.

**FIGURE 5:**
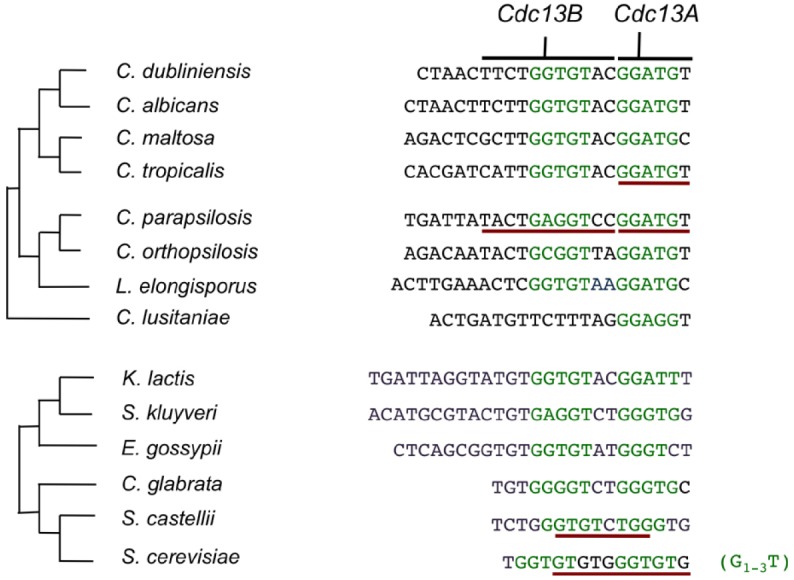
**The telomere repeat units of Saccharomycotina yeast and the putative or experimentally determined Rap1 and Cdc13 target sites.** The phylogeny of *Candida spp.* and that of *Saccharomyces* and *Kluyveromyces spp.* are displayed separately along with the telomere repeat unit in each species. The putative Rap1 half-sites are displayed in green and the nucleotides that have been experimentally shown to contact Cdc13 or be required for Cdc13 binding are underlined in dark red. Note that *C. lusitaniae* has an unusual telomere repeat unit that carry just one obvious candidate half site for Rap1.

First, the utilization of a pair of DBDs, either as parts of the same polypeptide or a dimeric complex, is probably advantageous (Figure 5). A domain with a short DNA target site may be capable of forming only a low affinity complex; incorporating two low affinity interactions in a single complex can substantially increase the overall affinity. The two-domain arrangement can also offer added flexibility to the system: variations in the spacing between the “half sites” are readily accommodated by two DBDs that can be flexibly positioned to each other. As illustrations, one can point to Rap1s in Saccharomycotina yeast, which have two Myb motifs and bind DNA with high affinity. In contrast, human and *S. pombe* Rap1s have just a single Myb motif and exhibit little or no DNA-binding activity. In addition, the apparent variations in the spacing between the Rap1 half sites in different organisms [e.g., 8 bp in *S. cerevisiae* and 7 bp in *C. albicans* (center-to-center distance)] are consistent with adaptions involving altered dispositions between the two Myb motifs (Figure 5). With regard to the Cdc13 family members, the utilization of two sets of protein–DNA contacts for high affinity binding is not universal. While *Candida* Cdc13 dimers probably all require two sets of DBD–DNA interactions to bind stably to G-tails, *S. cerevisiae* Cdc13 (despite forming dimers) binds G-tail with exceptionally high affinity using just one DBD–DNA interaction. This impressive feat of *Sc*Cdc13 is accomplished by expanding the typical OB-DNA interface through the acquisition of an extended and structurally well-defined L23. That is, rather than adding a second set of protein–DNA interaction, *Sc*Cdc13 was able to drastically expand the first set to enhance binding affinity. *S. castellii* Cdc13, another 4-OB fold Cdc13, also appears to need just one DBD–DNA interaction for high affinity binding. Whether this property applies to other large Cdc13s (e.g., *K. lactis* Cdc13) is an interesting question for future investigation.

A special case of achieving high affinity binding through two sets of protein–DNA interactions, employed by members of Cdc13 family only (specifically *Cp*Cdc13A and *Cp*Cdc13B), involves gene duplication and hetero-dimerization. Compared to homodimerization, this strategy has the advantage of allowing Cdc13 dimers to recognize a more complex target sequence made up of two distinct half sites. This advantage makes hetero-dimerization an especially adaptive strategy for the recognition of *Candida* telomere repeat units, which are long and complex.

The second common mechanistic feature that may enable ready adaption of Rap1 and Cdc13 to new telomere sequences is the ability of the DNA-binding surfaces of these proteins to undergo local conformational changes to accommodate different target sequence. This was implied by the huge number of Rap1 target sites in the *S. cerevisiae* genome and the very loose consensus sequence obtained for this protein. High resolution structural analyses of Rap1 bound to three target sequences provided amply illustration of this local flexibility at the molecular level (Taylor et al., 2000). In the case of Cdc13, there is no direct evidence yet for this local conformational flexibility. However, analysis of another ss telomere binding protein (TEBP from *Oxytricha nova*) revealed considerable tolerance of its binding surface to different sequences (Theobald and Schultz, 2003). Moreover, the intrinsically greater flexibility of ssDNA may further contribute to the ability of Cdc13 to accommodate sequence changes. An illustration of this, uncovered by investigation of *on*TEBP, is termed nucleotide shuffling, which involves the extrusion of a nucleotide away from the protein surface, and thus an alteration in the registry of the DNA (Theobald and Schultz, 2003). This phenomenon can conceivably allow insertional mutations in telomere DNA to be easily accommodated by Cdc13. Thus, both sequence-specific ss and dsDNA-binding proteins can exhibit limited versatility in binding multiple target sequences. Nevertheless, as noted earlier, promiscuous binding of telomere proteins to non-telomeric sites would probably be highly detrimental to cell physiology. Thus, limited versatility of Rap1 and Cdc13 in sequence-specific recognition probably is reflective of a finely calibrated evolutionary compromise.

### Conflict of Interest Statement

The authors declare that the research was conducted in the absence of any commercial or financial relationships that could be construed as a potential conflict of interest.
